# Combined Oral Contraceptives Bringing to Light May-Thurner Syndrome

**DOI:** 10.7759/cureus.22839

**Published:** 2022-03-04

**Authors:** Dina Alnabwani, Nagapratap Ganta, Smriti Kochhar, Veera Jayasree Latha Bommu, Reham Majzoub, Sharon Hechter, Priya Patel, Pramil Cheriyath

**Affiliations:** 1 Internal Medicine, Hackensack Meridian Ocean University Medical Center, Brick, USA; 2 Medicine, Hackensack Meridian Ocean University Medical Center, Brick, USA; 3 Medicine, Rowan University School of Osteopathic Medicine, Stratford, USA; 4 Internal Medicine, University of Texas Rio Grande Valley-Doctors Hospital at Renaissance, Edinburg, USA

**Keywords:** may-thurner syndrome, venous insufficiency, deep vein thrombosis, iliac vein thrombosis, oral contraceptives

## Abstract

May-Thurner syndrome (MTS) is a relatively uncommon clinical condition characterized by venous blockage in the left lower leg. Compression of the left common iliac vein by the right common iliac artery and the underlying vertebral body causes obstruction. We report a case of MTS with extensive venous thrombosis in a 44-year-old female who remained clinically silent until she used combined oral contraceptive pills (OCPs) for more than three years.

## Introduction

May-Thurner syndrome (MTS) is a commonly known congenital abnormality of the vascular system where the iliac artery compresses the vein. MTS usually has an asymptomatic presentation but in symptomatic patients, the presentation of MTS includes left lower extremity deep vein thrombosis (DVT), swelling, pain, venous claudication, ulcerations, and varicose veins. Rare symptoms include phlebitis and phlegmasia cerulea dolens [1]. Virchow's triad can be used to describe the etiology and assess the risk of thrombosis, especially of DVT, which consists of stasis, vessel damage, and hypercoagulability [2]. All these three criteria were met in this unique case of a 44-year-old female currently using combined oral contraceptive pills (OCPs) who presented with left leg pain secondary to MTS.

## Case presentation

A 44-year-old female presented to the emergency room (ER) with left lower back pain with associated discoloration and swelling for the past two days. She described the pain as a constant pressure that radiated to the left leg and groin. The patient stated her left leg feels heavy but denied any associated pain. The patient stated she had been experiencing a headache but attributed that to her anxiety. The patient stated increased urinary frequency but denied any burning with urination. The pain was worse in supine position but nothing helped relieve it. Her vitals were unremarkable other than a heart rate of 107 beats per minute. She presented to the ER two years ago with a similar complaint but the Doppler ultrasound was negative for any significant findings and was discharged without any further workup. On a physical examination, she was found to be tachycardic with regular rhythm and swelling of the proximal interphalangeal (PIP) joint of the third digit on the right hand. There was increased tenderness in the sacroiliac joint but no midline spinal tenderness.

The patient stated that the first day of her last menstrual period (LMP) was two days ago. Her past medical history is rheumatoid arthritis (RA) for which she takes acetaminophen (Tylenol) occasionally. Her other medications include levonorgestrel-ethinyl estradiol 0.1-20 mg tablets once a day since three and half years after the birth of her third baby. She is a lifetime non-smoker, occasional alcohol use but no recreational drug usage. Both the parents are alive with no medical conditions. 

**Figure 1:**
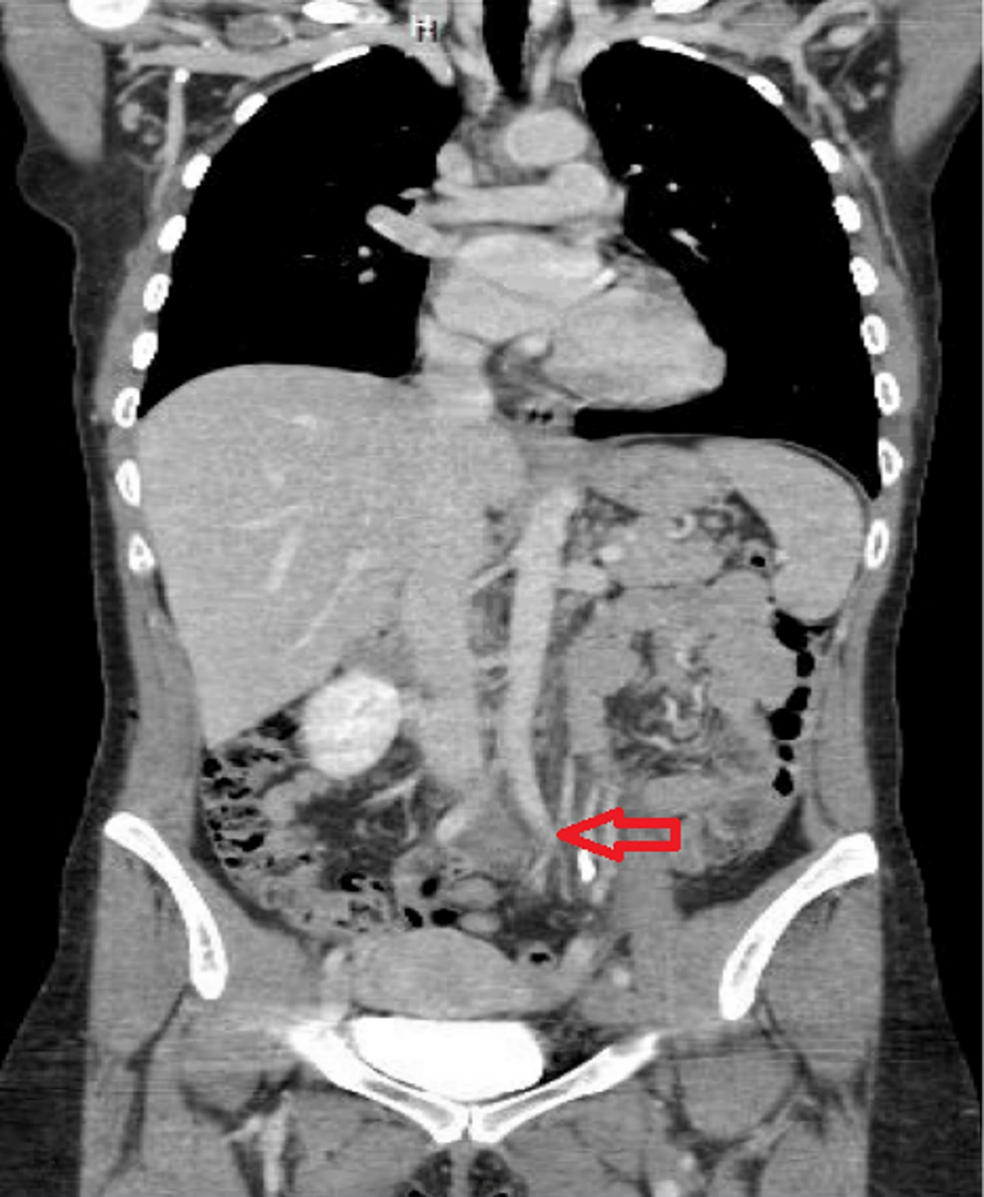
Computed tomography (CT) chest, abdomen, and pelvis with intravenous contrast The left common iliac vein, left external iliac vein, and sections of the left internal iliac vein were all found to be thrombosed (arrow).

Her labs showed an increased white blood cell count of 13.6 × 10^3^/uL (4.5-11.0 × 10^3^/uL) with no other significant abnormality on complete blood count (CBC), comprehensive metabolic panel (CMP), and coagulation studies. Doppler ultrasound of the left lower extremity showed an acute occlusive DVT identified from the left common femoral to the left popliteal vein. Occlusive thrombosis is also noted at the left peroneal and posterior tibial vein. The left common iliac vein, left external iliac vein, and sections of the left internal iliac vein were all found to be thrombosed on computed tomography (CT) scan of the chest, abdomen, and pelvis with intravenous contrast (Figure 1).

**Figure 2:**
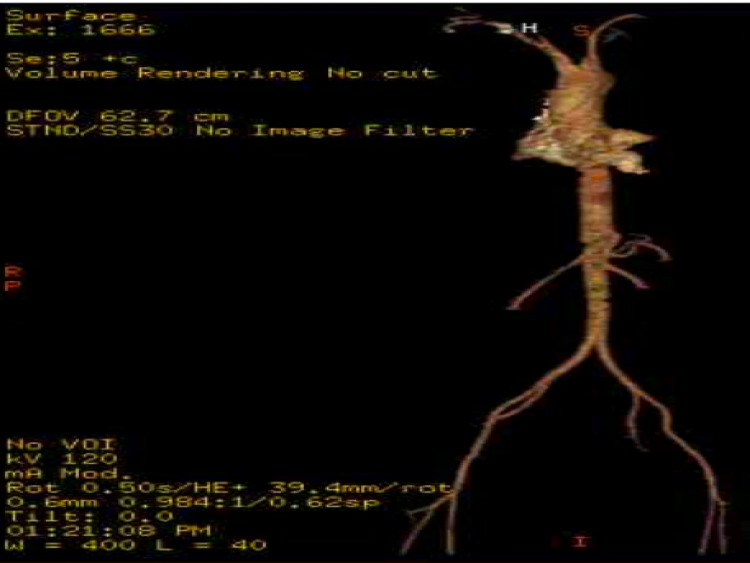
Three-dimensional computed tomography (CT) angiogram of chest, abdomen, and pelvis

The upper venous system of the left groin was thrombosed. The results were in line with MTS. Angiograms of the chest, abdomen, and pelvis revealed diffuse enlargement/heterogeneity covering the entire left common iliac vein, the left external iliac vein, and limited visualization of the left internal iliac vein on a three-dimensional CT scan (Figure 2). Findings were consistent with extensive thrombus. 

There was compression of the left common iliac vein by the right common iliac artery. She was started on enoxaparin 1 mg/kg subcutaneously every 12 hours. Later, the patient underwent mechanical venous thrombectomy with a left iliac vein stent without complications. She has discharged two days later on ​​apixaban 10 mg for seven days followed by 5 mg daily and aspirin 81 mg daily. Levonorgestrel-ethinylestradiol was discontinued.

## Discussion

May and Thurner, in 1957, identified an anatomic variation in which the right iliac artery compressed the left iliac vein, resulting in an elevated frequency of left iliofemoral DVT [3]. In a study of 50 patients, the prevalence of hemodynamically significant lesions (i.e., >50% stenosis) from a CT scan was approximately 25%. This study also found females are more significantly prone to MTS than males [4]. 

Chronic pulsatile stimulation from the overlaying common iliac artery irritates the endothelium of the left iliofemoral vein, resulting in the formation of bands, which are commonly referred to as spurs, in the pathophysiology of MTS. The majority of patients are asymptomatic because venous collaterals form to maintain blood flow continuity or the obstruction is not severe. It can only cause a DVT if there are transitory risk factors present, like as surgery, pregnancy, or post-partum [5]. 

In our patient, the Virchow's triad of clot formation was met, stasis or turbulent flow in this patient was due to thrombosis of the left common iliac vein, endothelial damage was due to the formation of bands and hypercoagulability is due to the oral contraceptives that the patient was started three years ago. OCPs are made up of both estrogen and progestin components in it. Estrogen in OCPs can influence gene transcription of many proteins, including clotting factor proteins, which can lead to an increase in plasma concentrations of these clotting factors. Higher estrogen doses seem to increase the risk of venous thrombus development [6]. The patient was on day two of her menstrual period when she started a new pack of oral contraceptive pills which are high in estrogen. The higher coagulability with increased compression leads to the symptomatic presentation in our patient. 

MTS should be considered as a possibility in young women who arrive with acute unilateral left limb edema and a notable size disparity between the limbs [1]. The clinical probability of DVT is determined using the Wells score and d-dimer in the initial workup for a patient with lower extremity edema. Patients with a moderate-to-high risk of lower extremity swelling should have a venous duplex ultrasound, which is the most reliable test for evaluating lower extremity swelling [7]. For patients suspected of having MTS, but without the presence of concomitant DVT on initial studies, additional noninvasive venous imaging (reflux duplex ultrasound with or without CT or magnetic resonance (MR) venography) may be useful [7]. If the clinical suspicion persists but non-invasive imaging fails to diagnose MTS, more invasive diagnostic modalities such as catheter-based venography and intravascular ultrasound can be used [7]. 

Compression stockings are used to treat MTS in the absence of DVT and with no or mild symptoms. In the absence of DVT, but with moderate-to-severe symptoms of chronic venous insufficiency, angioplasty, and stenting of the afflicted segment should be used to reduce the severity of the stenotic venous lesion. This treatment may not be sufficient in the long run because it is linked to significant recurrence rates [8]. Therapeutic anticoagulation should be used in patients who have a DVT with MTS. Following that, catheter-directed thrombolysis or pharmaco-mechanical thrombolysis are used to reduce the volume of thrombus, intravascular ultrasound (IVUS) is used to evaluate for underlying intrinsic venous stenosis, and if present, angioplasty, stenting of the diseased iliocaval segment is performed [7]. As in the instance of the above patient, who was treated with mechanical venous thrombectomy and left iliac vein stenting after receiving thrombolysis with a therapeutic dose of enoxaparin. She was discharged after five days of apixaban and daily aspirin.

## Conclusions

This case report talks about the clinical presentation, diagnosis, and possible treatments of MTS. Our patient was treated with mechanical venous thrombectomy and left iliac vein stenting after receiving thrombolysis with a therapeutic dose of enoxaparin. MTS is an often missed diagnosis as seen in our patient and therefore, physicians who treat patients with unilateral left leg pain presenting with features of venous insufficiency with or without DVT should rule out MTS.
